# Supplementary material for: Genome-wide association study in frontal fibrosing alopecia identifies four susceptibility loci including HLA-B*07:02
Source: Nat Commun. 2019 Mar 8;10:1150. doi: 10.1038/s41467-019-09117-w (PMC6408457; doi:10.1038/s41467-019-09117-w)
Supplement: Supplementary file 1 — Supplementary Information [file 41467_2019_9117_MOESM1_ESM.pdf]

Supplementary Information

Genome-wide association study in frontal fibrosing alopecia identifies four susceptibility loci and implicates *HLA-B*07:02* as the major genetic risk factor

Tziotzios, C., et al.

Supplementary Note 1

Frontal fibrosing alopecia (FFA) is a rare dermatological disorder associated with scarring, inflammation and irreversible hair loss affecting almost exclusively women of postmenopausal age.[1] Despite the paucity of epidemiologic estimates, it is widely accepted that the prevalence of FFA has been increasing worldwide since first reported in 1994 and it now represents the most common scarring alopecia in dermatology consultations, often attracting the descriptor epidemic for hair clinics.[2] Affected individuals report arm and leg hair loss to predate the eyebrow, facial and scalp involvement, with the latter becoming clinically evident and coinciding with oestrogen decline, whether menopausal or iatrogenic.[1] Oestrogen decline seems to be a key biochemical event in disease pathogenesis, as it heralds disease progression, possibly via immunomodulatory and profibrotic effects.[1] Segregation of FFA in first degree relatives and concordance in monozygotic twins provided a foundation for the notion that there is a genetic basis underlying disease pathogenesis, while the rapid increase in incidence was thought to be associated with (recent and female-specific) environmental triggers.[1] Yet, questionnaire-based studies interrogating topical cosmetic products, which are mostly not systemically absorbed, have failed to identify culprits with statistical significance.[3]

Male FFA is extremely rare: in our own cohort of 1, 440 cases, only 26 male cases were identified representing 1.8% of the total, which is in keeping with the fact that cases or small case series are rarely reported.[4,5] Hormonal aberrations, notably hypogonadism, have been thought to be associated with the disease, in line with published case series.[4, 5] Having considered the small sample size of male cases and the paucity of sufficient clinical phenotyping, biochemical and genetic data, we have focused on female patients, although it should be acknowledged that male disease does warrant separate study.

Supplementary figures

Supplementary Figure 1 Histological evaluation of clinically normal parietal skin from each healthy control (haematoxylin-eosin stain)

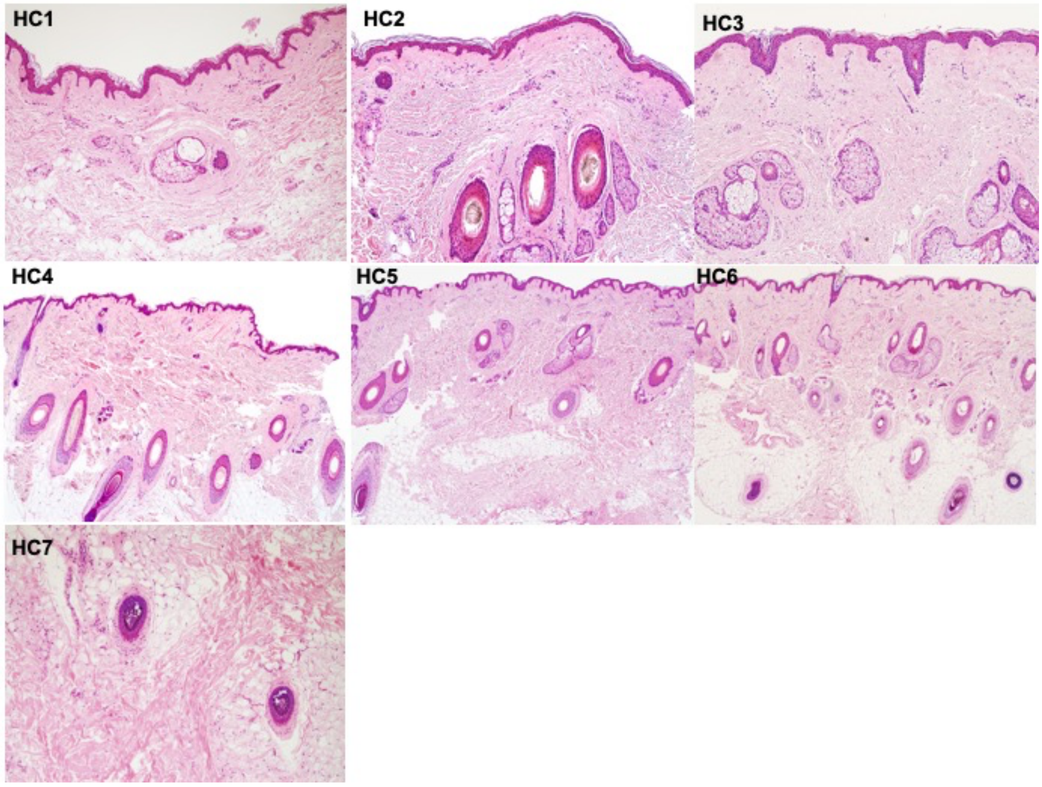

Supplementary Figure 2 Bioinformatic analysis workflow from sequencer output to differential gene expression

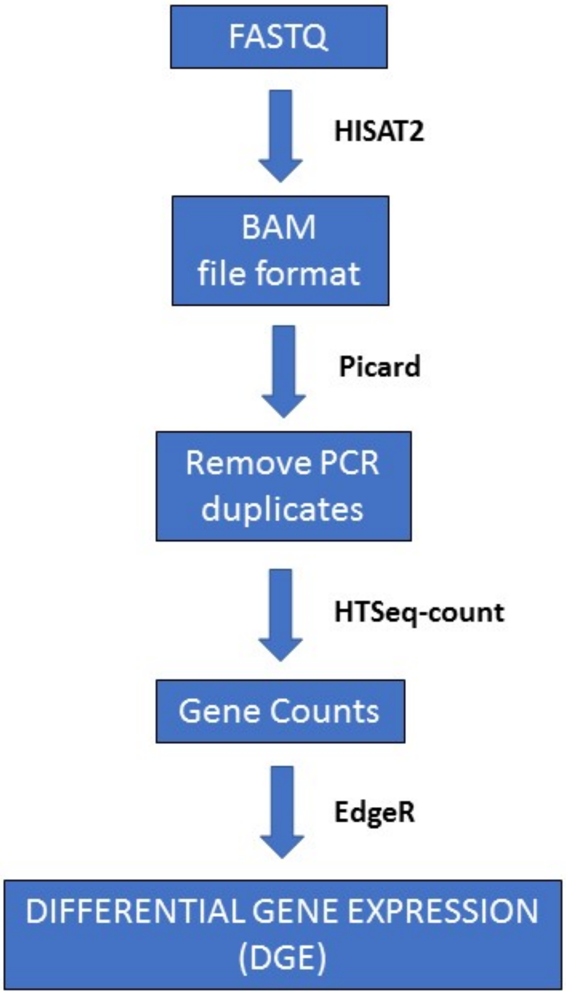

Supplementary Figure 3 Quantile-Quantile (Q-Q) plots of GWA results for UK cohort (A), Spanish cohort (B) and Meta-analysis (C)

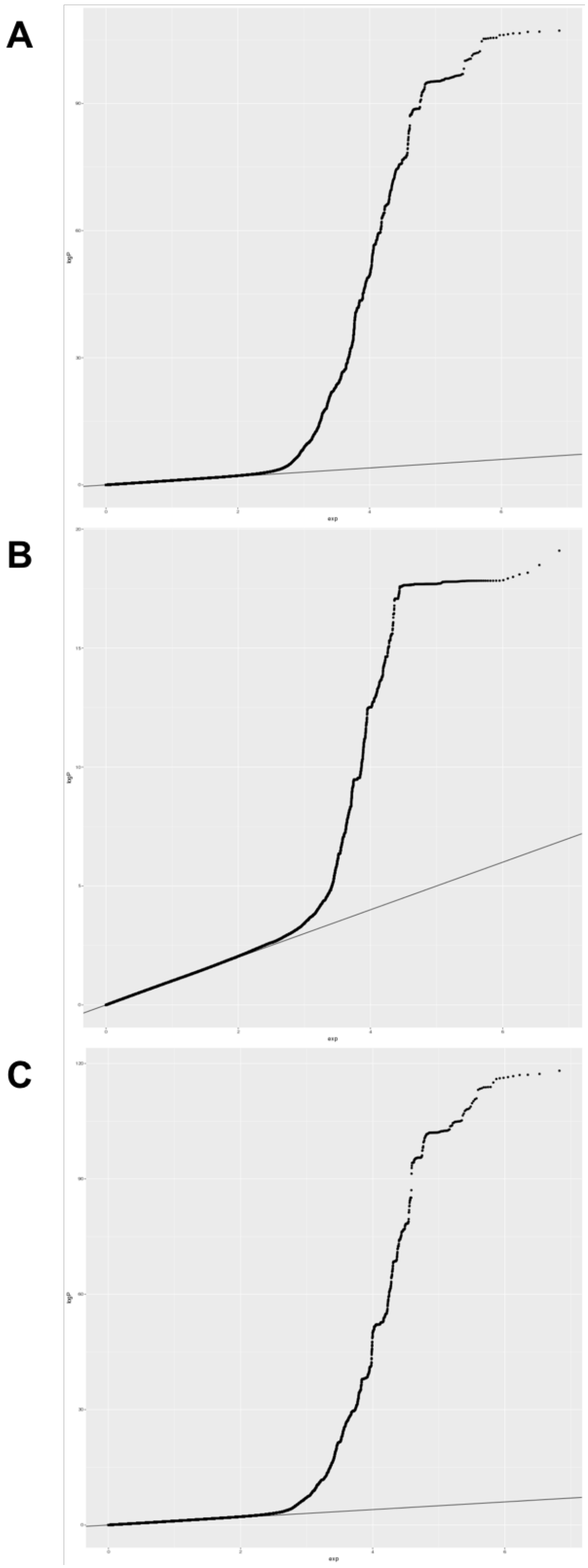

Supplementary Figure 4 Stepwise conditional testing at HLA locus

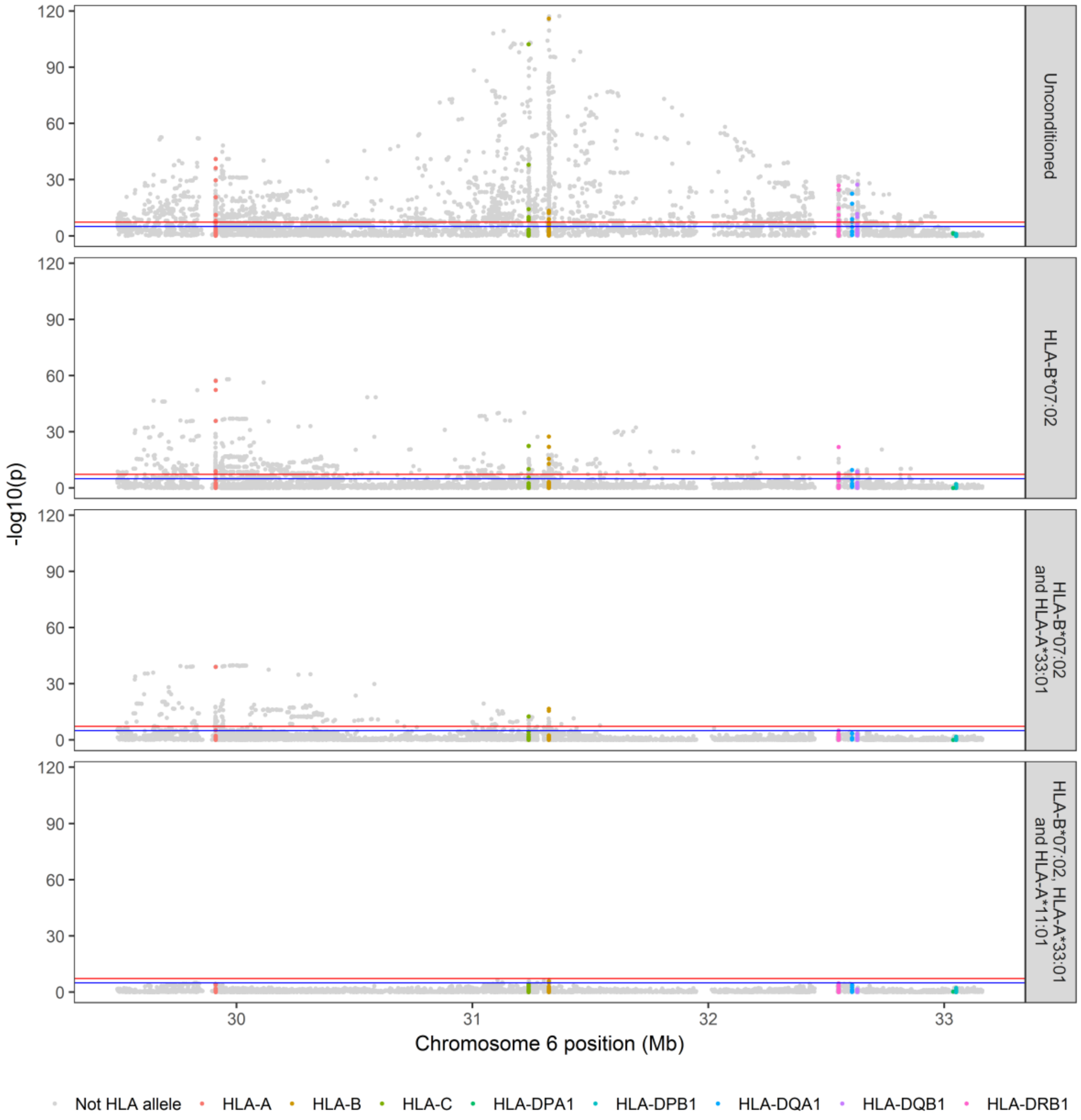

Supplementary Figure 5 Colocalisation of the FFA association signal (regional plot in top panel) with *SEMA4B* eQTL in sun-exposed skin from the GTEx database (bottom panel)

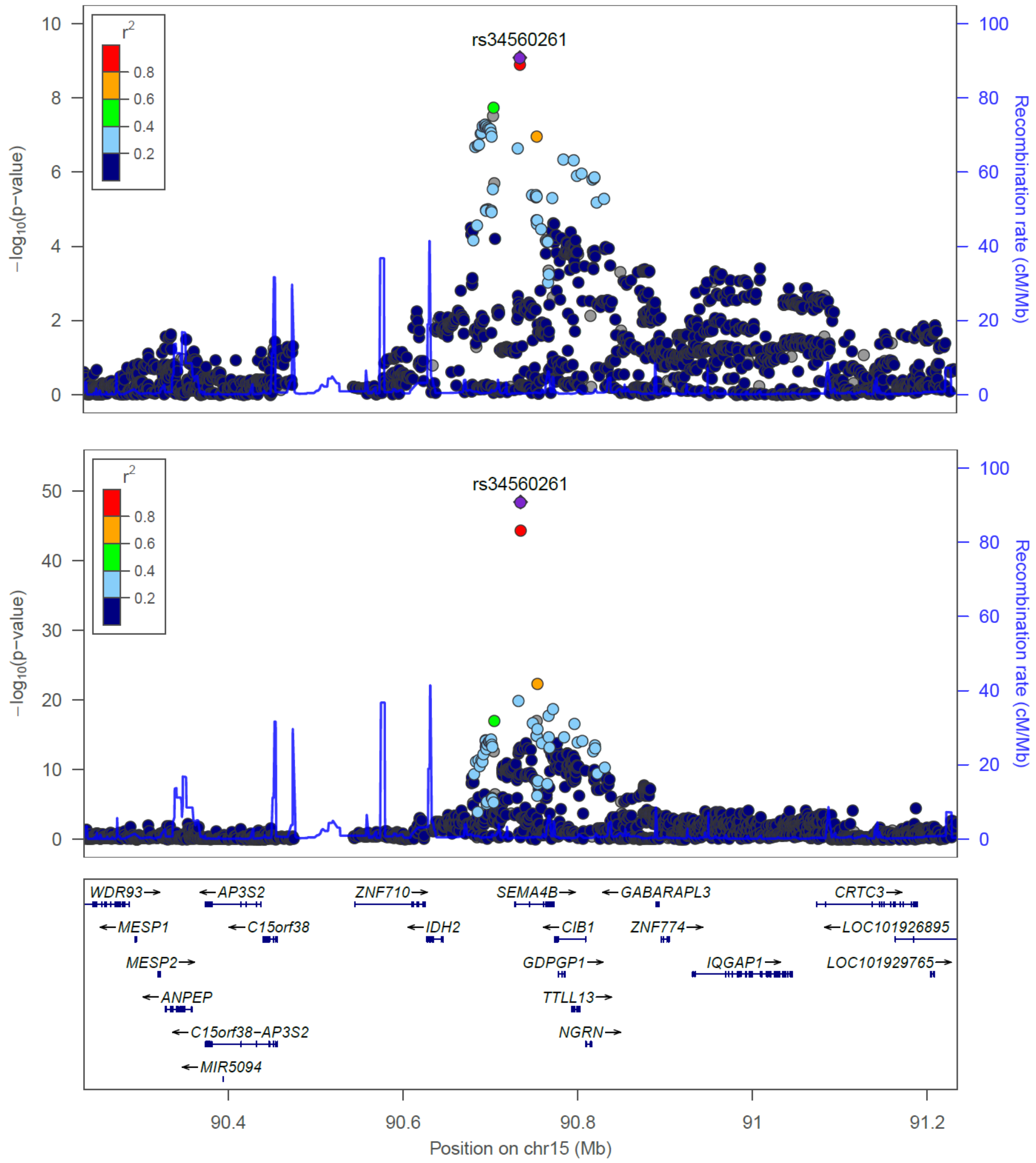

Supplementary Figure 6 HeatMap with two-way hierarchical clustering illustrating metabolomic differences between cases and controls for top 30 metabolites; each coloured cell on the map corresponds to a concentration value with samples in rows and compounds in columns

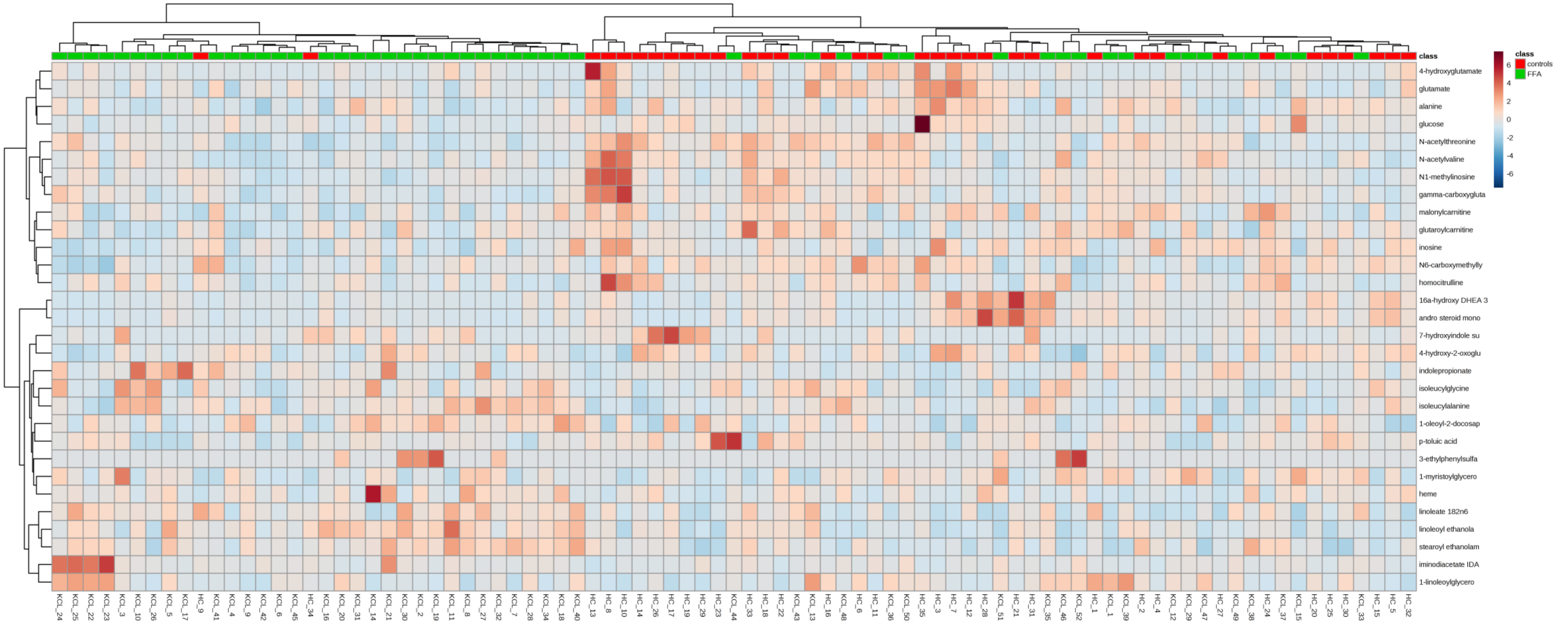

Supplementary Figure 7 Two-way hierarchical clustering RNAseq HeatMap generated based on the top 120 differentially expressed genes of 14 human scalp tissue samples (7 healthy controls, *HC*; and 7 FFA cases, *Case*); up- and down-regulated genes are depicted in yellow and green and red, respectively, and colour intensity corresponds to magnitude

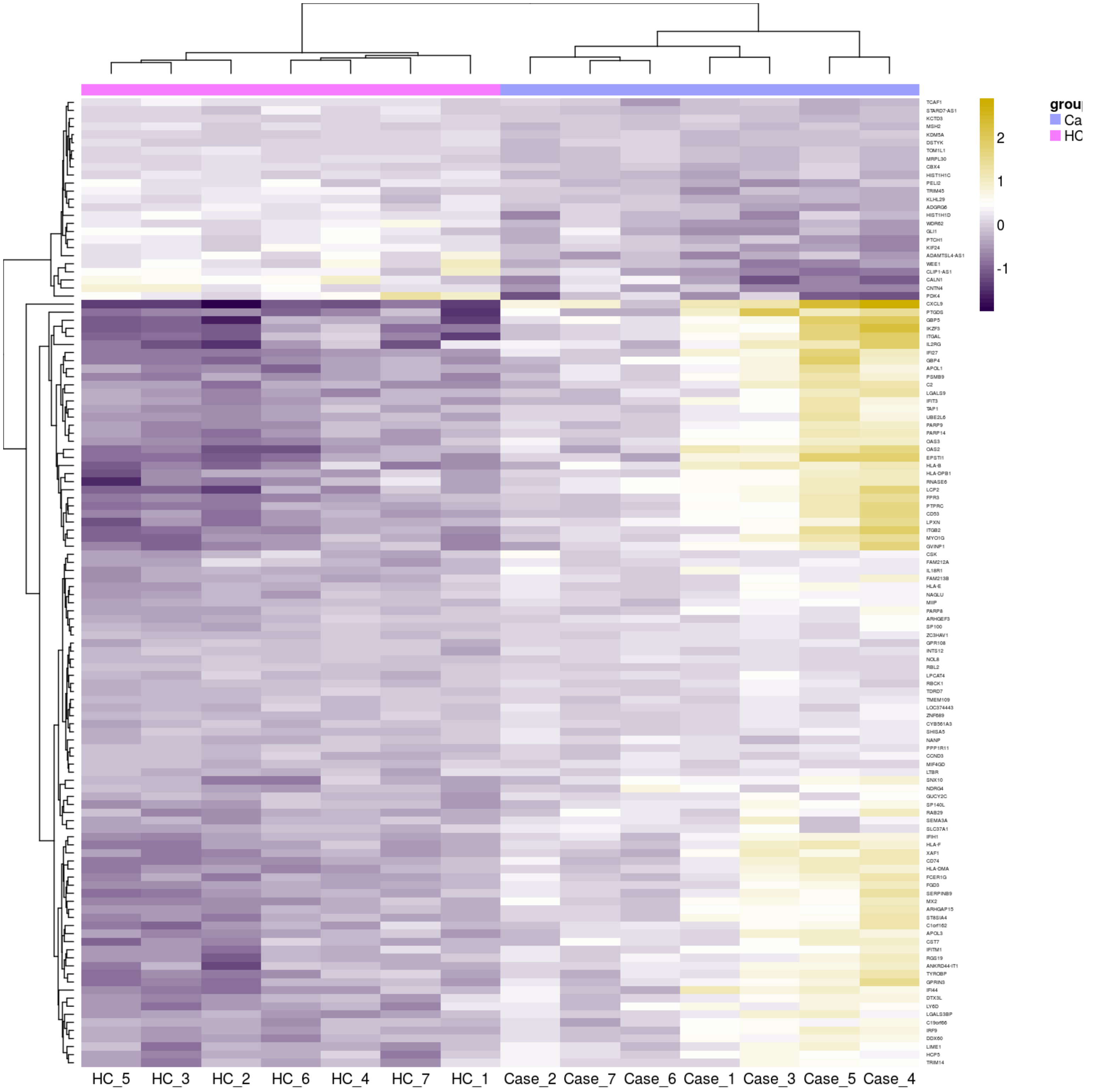

Supplementary tables

Supplementary Table 1 Inclusion criteria for transcriptomic analysis

**Inclusion criteria**
Gender: female
Ethnicity: Caucasian
Age: >51 years
Clinical and histological evidence of active lichenoid inflammation (cases)
Clinical and histological evidence of absence of lichenoid inflammation (controls)
Immunosuppressant and anti-inflammatory treatment-(topical and systemic) naïve (cases and controls)
Absence of concomitant dermatosis in clinical history (cases)
Absence of any dermatosis in clinical history (controls)

Supplementary Table 2 Population confounding bias metrics for each study

	**UK cohort**	**Spanish cohort**	**Meta-analysis**
$\lambda_{GC\ (MAF>0.05)}$	1.03	1.03	1.03
**LD score regression intercept**	1.005	1.013	1.006

Supplementary Table 3 Suggestive significant loci (Meta-analysis)

							UK		Spanish		Meta-analysis		
CHR	SNP ID	RA	PA	Position (hg19)	RAF_{Cases}	$RAF_{Controls}$	OR (95% CI)	P	OR (95% CI)	P	OR (95% CI)	P	P_{Het}
10	rs117687547	T	C	12, 504, 313	0.049	0.031	1.83 (1.41-2.40)	$8.11x10^{-6}$	1.68 (0.57-4.97)	0.34	1.83 (1.41-2.36)	$5.14x10^{-6}$	0.88
10	rs112198986	T	C	24, 576, 259	0.056	0.037	1.94 (1.49-2.52)	$7.40x10^{-7}$	0.92 (0.38-2.21)	0.85	1.82 (1.42-2.34)	$2.75x10^{-6}$	0.11
12	rs111463574	C	T	95, 399, 358	0.949	0.915	1.69 (1.33-2.14)	$1.52x10^{-5}$	1.93 (1.11-3.36)	0.02	1.72 (1.38-2.14)	$1.06x10^{-6}$	0.66
13	rs10507508	G	A	42, 969, 782	0.069	0.044	1.62 (1.29-2.03)	$3.05x10^{-5}$	2.16 (1.08-4.34)	0.03	1.66 (1.34-2.06)	$3.55x10^{-6}$	0.44
16	rs6540122	T	C	87, 991, 361	0.199	0.160	1.39 (1.20-1.60)	$5.74x10^{-6}$	1.12 (0.81-1.56)	0.49	1.34 (1.18-1.53)	$9.06x10^{-6}$	0.25
17	rs8065764	C	T	21, 006, 462	0.916	0.877	1.60 (1.32-1.95)	$2.49x10^{-6}$	1.36 (0.86-2.14)	0.19	1.56 (1.30-1.87)	$1.27x10^{-6}$	0.51
17	rs12951836	G	A	28, 410, 277	0.573	0.507	1.26 (1.13-1.41)	$4.38x10^{-5}$	1.38 (1.07-1.79)	0.01	1.28 (1.15-1.41)	$2.44x10^{-6}$	0.51
17	rs112659862	C	T	71, 290, 983	0.015	0.005	3.26 (1.86-5.70)	$3.57x10^{-5}$	16.85 (2.01-141.52)	0.01	3.62 (2.11-6.22)	$3.17x10^{-6}$	0.14
19	rs79459566	T	C	13, 320, 504	0.062	0.040	1.82 (1.43-2.31)	$1.17x10^{-6}$	0.92 (0.43-2.00)	0.84	1.71 (1.36-2.15)	$4.66x10^{-6}$	0.10
2	rs13032164	C	G	151, 760, 452	0.872	0.836	1.43 (1.21-1.68)	$2.73x10^{-5}$	1.38 (0.95-2.00)	0.09	1.42 (1.22-1.65)	$6.05x10^{-6}$	0.88
2	rs78504246	A	G	213, 746, 962	0.013	0.005	3.52 (1.95-6.34)	$2.89x10^{-5}$	4.03 (0.70-23.09)	0.12	3.57 (2.04-6.23)	$8.12x10^{-6}$	0.89
2	rs6731286	T	A	62, 839, 819	0.714	0.658	1.32 (1.17-1.49)	$6.05x10^{-6}$	1.28 (0.97-1.70)	0.08	1.32 (1.18-1.47)	$1.24x10^{-6}$	0.85
3	rs13078360	A	G	127, 074, 825	0.245	0.200	1.33 (1.16-1.52)	$3.18x10^{-5}$	1.50 (1.09-2.07)	0.01	1.35 (1.20-1.53)	$1.67x10^{-6}$	0.49
3	rs116806118	T	A	23, 165, 701	0.014	0.005	3.66 (2.03-6.60)	$1.52x10^{-5}$	2.41 (0.80-7.27)	0.12	3.34 (1.99-5.61)	$5.34x10^{-6}$	0.51
3	rs1461070	A	G	30, 741, 506	0.031	0.017	2.15 (1.53-	$8.23x10^{-6}$	2.26 (0.88-	0.09	2.16 (1.57-	$1.84x10^{-6}$	0.92

							3.00)		5.80)		2.96)		
3	rs114108912	C	G	74, 464, 791	0.023	0.010	2.47 (1.61-3.79)	8.2×10^{-5}	2.61 (0.81-8.41)	0.11	2.49 (1.67-3.72)	8.29×10^{-6}	0.93
4	rs183175	C	G	5, 428, 385	0.703	0.646	1.34 (1.19-1.52)	2.32×10^{-6}	1.03 (0.77-1.37)	0.84	1.29 (1.15-1.44)	9.61×10^{-6}	0.10
5	rs73062921	A	T	16, 859, 464	0.148	0.113	1.37 (1.16-1.62)	0.00017	1.63 (1.11-2.39)	0.01	1.41 (1.21-1.64)	8.84×10^{-6}	0.42
5	rs10045403	G	A	96, 147, 733	0.324	0.266	1.28 (1.14-1.45)	3.49×10^{-5}	1.65 (1.22-2.22)	0.001	1.33 (1.19-1.48)	4.15×10^{-7}	0.13
6	rs148661203	T	C	10, 220, 442	0.017	0.007	2.82 (1.69-4.70)	7.12×10^{-5}	4.17 (1.05-16.49)	0.04	2.96 (1.83-4.78)	9.31×10^{-6}	0.60
6	rs34097647	T	C	151, 430, 073	0.2415	0.197	1.33 (1.16-1.52)	2.99×10^{-5}	1.58 (1.13-2.22)	0.007	1.36 (1.20-1.55)	1.08×10^{-6}	0.35
6	rs142366299	T	C	52, 179, 367	0.025	0.012	2.62 (1.71-4.01)	8.63×10^{-6}	1.65 (0.77-3.50)	0.20	2.34 (1.62-3.40)	6.42×10^{-6}	0.29
7	rs2021162	G	A	126, 528, 455	0.326	0.276	1.30 (1.16-1.47)	1.47×10^{-5}	1.20 (0.89-1.60)	0.23	1.29 (1.15-1.44)	7.98×10^{-6}	0.59
7	rs112115472	C	T	13, 645, 859	0.984	0.963	2.62 (1.72-4.00)	7.17×10^{-6}	2.90 (1.07-7.82)	0.04	2.66 (1.81-3.93)	7.31×10^{-7}	0.86
7	rs6975452	T	A	3, 160, 511	0.559	0.504	1.25 (1.12-1.40)	9.59×10^{-5}	1.52 (1.17-1.99)	0.002	1.29 (1.16-1.43)	1.64×10^{-6}	0.18
7	rs7806494	A	T	3, 893, 629	0.766	0.723	1.36 (1.19-1.56)	7.41×10^{-6}	1.22 (0.90-1.64)	0.20	1.34 (1.18-1.51)	4.06×10^{-6}	0.50
9	rs2773871	G	A	124, 873, 393	0.836	0.802	1.39 (1.19-1.62)	2.26×10^{-5}	1.28 (0.94-1.75)	0.12	1.37 (1.19-1.57)	7.35×10^{-6}	0.63
9	rs277580	G	A	34, 771, 875	0.884	0.837	1.54 (1.30-1.82)	4.00×10^{-7}	1.03 (0.70-1.50)	0.88	1.44 (1.24-1.68)	2.59×10^{-6}	0.06

***** RA, risk allele; PA, protective allele; RAF, risk allele frequency; OR, odds ratio; RAF, risk allele frequency; CI, confidence interval

Supplementary Table 4 Imputed HLA alleles (A); and SNPs (B) for UK, Spain and Meta-analysis (AF: allele frequency; RAF: risk allele frequency)

A

		UK Cohort			Spanish Cohort			Meta-analysis		Original results (unconditional meta-analysis)	
**Allele**	**Alleles conditioned on**	**AF case/ctrl**	**OR (95% CI)**	**P**	**AF case/ctrl**	**OR (95% CI)**	**P**	**OR (95% CI)**	**P**	**OR (95% CI)**	**P**
HLA-B*07:02	-	0.411/ 0.141	5.34 (4.60-6.20)	$1.09{\times}10^{-106}$	0.223/ 0.078	4.38 (2.88-6.65)	$5.18{\times}10^{-12}$	5.22 (4.53-6.01)	$9.44{\times}10^{-117}$	-	-
HLA-A*33:01	HLA-B*07:02	0.040/ 0.006	16.17 (10.50-24.88)	$1.14{\times}10^{-36}$	0.207/ 0.020	28.71 (14.83-55.58)	$2.25{\times}10^{-23}$	19.19 (13.38-27.54)	$6.91{\times}10^{-58}$	10.18 (7.27-14.24)	$1.02{\times}10^{-41}$
HLA-A*11:01	HLA-B*07:02 and HLA-A*33:01	0.142/ 0.059	3.68 (3.00-4.52)	$1.52{\times}10^{-35}$	0.140/ 0.071	3.40 (1.97-5.87)	$1.05{\times}10^{-5}$	3.64 (3.01-4.42)	$9.28{\times}10^{-40}$	2.66 (2.25-3.14)	$1.90{\times}10^{-30}$

B

		UK Cohort			Spanish Cohort			Meta-analysis		Original results (unconditional meta-analysis)		
**SNP**	**SNPs conditioned on**	**RAF case/ctrl**	**OR (95% CI)**	**P**	**RAF case/ctrl**	**OR (95% CI)**	**P**	**OR (95% CI)**	**P**	**OR (95% CI)**	**P**	**Correlated HLA allele***
rs2523616	-	0.474/ 0.189	2.78 (2.47-3.15)	$5.04\text{x}10^{-61}$	0.448/ 0.162	3.14 (2.33-4.23)	$5.13\text{x}10^{-14}$	4.73 (4.15-5.39)	$7.6\text{x}10^{-119}$	-	-	HLA-B*07
rs62388754	rs2523616	0.141/ 0.058	3.54 (2.90-4.33)	$5.46\text{x}10^{-35}$	0.140/ 0.064	3.47 (2.09-5.75)	$1.49\text{x}10^{-6}$	3.53 (2.93-4.25)	$4.54\text{x}10^{-40}$	2.76 (2.33-3.27)	$5.06\text{x}10^{-32}$	HLA-A*11:01
rs147324178	rs2523616 and rs62388754	0.417/ 0.144	2.89 (2.22-3.75)	$2.15\text{x}10^{-15}$	0.238/ 0.086	1.32 (0.78-2.25)	0.2991	2.48 (1.96-3.14)	$3.65\text{x}10^{-14}$	5.25 (4.56-6.04)	$5.61\text{x}10^{-118}$	HLA-B*07
rs7749944	rs2523616, rs62388754 and rs147324178	0.040/ 0.006	15.70 (9.75-25.3)	$8.51\text{x}10^{-30}$	0.206/ 0.021	27.7 (12.5-61.2)	$2.19\text{x}10^{-16}$	18.24 (12.1-27.4)	$3.18\text{x}10^{-44}$	10.06 (7.20-14.1)	$7.92\text{x}10^{-42}$	HLA-A*33:01

* Indicates classical 2- or 4-digit HLA allele in strongest linkage disequilibrium with each SNP (R^2 estimated from SNP/HLA allele dosages based on Pearson correlation across combined UK and Spanish cohorts).

Supplementary Table 5 Bayesian fine-mapping of the three independent FFA association signals

Band	Implicated gene	N within 95% CI	Variant with highest posterior probability (V_{Top})	P_{Causal} for V_{Top}
2p22.2	*CYP1B1*	1	rs1800440	0.9833
8q24.22	*ST3GAL1*	12	rs760327	0.6798
15q26.1	*SEMA4B*	18	rs34560261	0.4262

Supplementary Table 6 Mann-Whitney U test for metabolomic case-control analysis; metabolites with individual p<0.05 are presented

Metabolite	$Median_{cases}$	$Median_{controls}$	Z score	P*
iminodiacetate (IDA)	1.07	0.96	-3.61	0.0003
andro steroid monosulfate (1)*	0.55	1.32	3.35	0.0008
glucose	0.97	1.04	3.35	0.0008
docosahexaenoate (DHA; 22:6n3)	1.08	0.79	-3.17	0.002
heme	1.18	0.62	-3.17	0.002
16a-hydroxy DHEA 3-sulfate	0.74	1.57	2.91	0.004
6-hydroxyindole sulfate	0.93	1.20	2.91	0.004
N6-carboxymethyllysine	0.92	1.23	2.91	0.004
p-toluic acid	0.88	1.14	2.91	0.004
1-stearoyl-2-linoleoyl-GPI (18:0/18:2)	1.09	0.87	-2.74	0.01
eicosapentaenoate (EPA; 20:5n3)	1.09	0.90	-2.74	0.01
isoleucylglycine	1.09	0.79	-2.74	0.01
S-adenosylhomocysteine (SAH)	1.03	0.84	-2.74	0.01
1-oleoyl-2-docosahexaenoyl-GPC (18:1/22:6)*	1.06	0.91	-2.74	0.01
3-ethylphenylsulfate	0.14	0.14	-2.58	0.01
1-palmityl-2-arachidonoyl-GPC (O-16:0/20:4)*	0.94	1.10	2.48	0.01
androstenediol (3beta, 17beta) disulfate (2)	0.90	1.09	2.48	0.01
N-acetylthreonine	0.93	1.12	2.48	0.01
phosphate	0.99	1.05	2.48	0.01
2-aminophenol sulfate	1.38	0.91	-2.30	0.02
allantoin	1.05	0.94	-2.30	0.02
cinnamoylglycine	1.24	0.89	-2.30	0.02
isoleucylalanine	1.10	0.86	-2.30	0.02
linoleate (18:2n6)	1.07	0.81	-2.30	0.02
N-palmitoylglycine	1.06	0.93	-2.30	0.02
1-linoleoylglycerol (18:2)	1.20	0.87	-2.30	0.02
oleate/vaccenate (18:1)	1.16	0.90	-2.30	0.02
stearoyl ethanolamide	1.07	0.96	-2.30	0.02
1-(1-enyl-palmitoyl)-2-arachidonoyl-GPC (P-16:0/20:4)*	0.98	1.08	2.04	0.04
2-arachidonoyl-GPE* (20:4)*	0.50	0.83	2.04	0.04
21-hydroxypregnenolone disulfate	0.88	1.24	2.04	0.04
3-indoxyl sulfate	0.89	1.17	2.04	0.04
alanine	0.96	1.06	2.04	0.04
1-arachidonoyl-GPE* (20:4)*	0.90	1.04	2.04	0.04
cortisol	0.94	1.12	2.04	0.04
ethylmalonate	0.89	1.16	2.04	0.04
gamma-carboxyglutamate	0.99	1.11	2.04	0.04
homocitrulline	0.89	1.19	2.04	0.04
inosine	0.84	1.11	2.04	0.04

N1-methylinosine	0.95	1.07	2.04	0.04
oleamide	0.87	1.27	2.04	0.04
p-cresol-glucuronide*	0.73	1.14	2.04	0.04
phenylalanine	0.99	1.06	2.04	0.04
pregnenolone sulfate	0.88	1.24	2.04	0.04
sucrose	0.90	1.46	2.04	0.04
taurocholenate sulfate	0.94	1.17	2.04	0.04
21-hydroxypregnenolone monosulfate (1)	0.50	0.50	2.00	0.05

*Bonferroni-adjusted values not displayed as none <0.05

Supplementary Table 7 Numbers of differentially Expressed Genes (DEGs) between cases and controls

Differentially Expressed Genes with FDR ≤ 0.05:	327
Differentially Expressed Genes with FDR ≤ 0.05 & LFC ≥ 1:	120
Differentially Expressed Genes UP FDR ≤ 0.05 & |LFC| ≥ 1:	83
Differentially Expressed Genes DOWN FDR ≤ 0.05 & |LFC| ≥ 1:	37

* LFC = log fold change; FDR = false discovery rate

Supplementary Table 8 Upregulated significantly differentially expressed genes (DEGs) between FFA cases and controls (FDR ≤ 0.05 & |LFC| ≥ 1) *

Genes	logFC	logCPM	LR	p value	FDR
CXCL9	2.98	5.17	51.42	$7.45x10^{-13}$	$9.70x10^{-9}$
GBP5	1.97	4.44	35.3	$2.83x10^{-9}$	$1.84x10^{-5}$
PSMB9	1.14	4.8	32.11	$1.46x10^{-8}$	$6.34x10^{-5}$
IFI27	1.41	5.79	29.54	$5.48x10^{-8}$	$1.25x10^{-4}$
ITGAL	1.71	4.09	28.21	$1.09x10^{-7}$	$1.63x10^{-4}$
PTGDS	1.82	4.88	27.49	$1.58x10^{-7}$	$1.94x10^{-4}$
GBP4	1.41	5.65	27.41	$1.64x10^{-7}$	$1.94x10^{-4}$
LCP2	1.43	3.87	26.95	$2.09x10^{-7}$	$2.27x10^{-4}$
C2	1.17	4.42	26.04	$3.35x10^{-7}$	$2.91x10^{-4}$
MYO1G	1.3	3.6	25.02	$5.66x10^{-7}$	$4.10x10^{-4}$
OAS2	1.51	4.61	24.3	$8.25x10^{-7}$	$5.38x10^{-4}$
LPXN	1.17	3.97	24.12	$9.07x10^{-7}$	$5.63x10^{-4}$
PARP14	1.01	7.48	23.96	$9.83x10^{-7}$	$5.82x10^{-4}$
EPSTI1	1.65	4.14	23.63	$1.17x10^{-6}$	$6.34x10^{-4}$
IKZF3	1.78	4.4	23.13	$1.51x10^{-6}$	$7.59x10^{-4}$
ITGB2	1.36	6.12	22.78	$1.82x10^{-6}$	$8.77x10^{-4}$
SASH3	1.71	3.86	22.44	$2.17x10^{-6}$	$9.73x10^{-4}$
CD74	1.05	9.12	21.85	$2.95x10^{-6}$	$1.20x10^{-3}$
IGLL5	2.95	3.77	21.66	$3.25x10^{-6}$	$1.25x10^{-3}$
PTPRCAP	1.47	3.62	21.44	$3.65x10^{-6}$	$1.33x10^{-3}$
APOL1	1.1	4.21	21.43	$3.68x10^{-6}$	$1.33x10^{-3}$
RNASE6	1.15	3.28	21.37	$3.79x10^{-6}$	$1.34x10^{-3}$
GVINP1	1.23	4.46	21.2	$4.14x10^{-6}$	$1.39x10^{-3}$
FPR3	1.14	4.75	21.18	$4.17x10^{-6}$	$1.39x10^{-3}$
CD53	1.22	4.34	20.88	$4.90x10^{-6}$	$1.49x10^{-3}$
GPRIN3	1.11	4.3	20.12	$7.26x10^{-6}$	$1.89x10^{-3}$
JCHAIN	2.08	4.1	19.22	$1.17x10^{-5}$	$2.53x10^{-3}$
PTPRC	1.16	6.47	18.92	$1.36x10^{-5}$	$2.82x10^{-3}$
LYZ	1.26	5.79	18.11	$2.08x10^{-5}$	$3.71x10^{-3}$
LGALS9	1.03	3.12	18.17	$2.02x10^{-5}$	$3.71x10^{-3}$
MMP9	2.47	5.26	18.01	$2.20x10^{-5}$	$3.72x10^{-3}$
PARVG	1.3	3.41	18.03	$2.17x10^{-5}$	$3.72x10^{-3}$
TYROBP	1	4.01	18.03	$2.17x10^{-5}$	$3.72x10^{-3}$
SPOCK2	1.33	4.54	17.59	$2.74x10^{-5}$	$4.46x10^{-3}$
IRF1	1.23	5.67	17.61	$2.71x10^{-5}$	$4.46x10^{-3}$
ITGAX	1.37	4.05	17.42	$3.00x10^{-5}$	$4.73x10^{-3}$
MX1	1.13	5.75	17.41	$3.02 x10^{-5}$	$4.73x10^{-3}$
NCKAP1L	1	5.48	17.32	$3.15x10^{-5}$	$4.78x10^{-3}$
FYB	1.19	5.03	17.04	$3.66x10^{-5}$	$5.36x10^{-3}$

VAV1	1.05	3.78	16.86	$4.02x10^{-5}$	$5.46x10^{-3}$
HCLS1	1.05	5.09	16.87	$4.01x10^{-5}$	$5.46Ex10^{-3}$
DOCK2	1.02	5.76	16.88	$3.98x10^{-5}$	$5.46x10^{-3}$
ZIC1	1.74	3.37	16.81	$4.14x10^{-5}$	$5.56x10^{-3}$
IL7R	1.59	4.96	16.7	$4.38x10^{-5}$	$5.65x10^{-3}$
ADAM28	1.27	3.9	16.71	$4.36x10^{-5}$	$5.65x10^{-3}$
CCL19	1.39	4.88	16.57	$4.69x10^{-5}$	$5.92x10^{-3}$
TRAF1	1.01	4.57	16.38	$5.19x10^{-5}$	$6.44x10^{-3}$
APOBEC3G	1.21	3.31	16.25	$5.56x10^{-5}$	$6.68x10^{-3}$
CORO1A	1.21	4.95	16.27	$5.48x10^{-5}$	$6.68x10^{-3}$
IRF8	1.29	4.52	16.08	$6.09x10^{-5}$	$7.15x10^{-3}$
CD84	1.05	5.14	16.05	$6.17x10^{-5}$	$7.15x10^{-3}$
IL2RG	1.53	3.02	16.02	$6.28x10^{-5}$	$7.18\ x10^{-3}$
SPP1	2.23	5.08	15.87	$6.79\ x10^{-5}$	$7.59x10^{-3}$
TRAF3IP3	1.14	3.33	15.83	$6.93x10^{-5}$	$7.59x10^{-3}$
JAK3	1.43	3.48	15.54	$8.06x10^{-5}$	$8.40x10^{-3}$
LAPTM5	1.24	5.69	15.39	$8.73x10^{-5}$	$8.70x10^{-3}$
PLAU	1.03	4.52	15.17	$9.83x10^{-5}$	$9.42x10^{-3}$
CLEC10A	1.02	4.47	15.18	$9.78x10^{-5}$	$9.42x10^{-3}$
SPN	1.01	4.42	15.07	$1.04x10^{-4}$	$9.59x10^{-3}$
CYTH4	1.04	4.17	15.05	$1.05x10^{-4}$	$9.60x10^{-3}$
SP140	1.22	3.24	14.98	$1.09x10^{-4}$	$9.92x10^{-3}$
TFEC	1.15	3.3	14.92	$1.12x10^{-4}$	$1.01x10^{-3}$
WDFY4	1.22	5.56	14.85	$1.17x10^{-4}$	$1.03x10^{-3}$
IKZF1	1.17	4.53	14.64	$1.30x10^{-4}$	$1.10x10^{-3}$
ITK	1.29	3.6	13.97	$1.86x10^{-4}$	$1.43x10^{-3}$
CMPK2	1.1	2.92	13.98	$1.85x10^{-4}$	$1.43\ x10^{-3}$
IFI30	1.16	5.4	13.94	$1.88\ x10^{-4}$	$1.44\ x10^{-3}$
SELE	1.57	3.87	13.61	$2.25x10^{-4}$	$1.59x10^{-3}$
PLEK	1.13	4.73	13.57	$2.29x10^{-4}$	$1.61x10^{-2}$
PLCB2	1.19	3.74	13.42	$2.49x10^{-4}$	$1.73x10^{-2}$
KLHL6	1.19	3.42	13.33	$2.61x10^{-4}$	$1.79x10^{-2}$
SLAMF8	1.1	3.03	13.07	$3.00x10^{-4}$	$1.95x10^{-2}$
SELPLG	1	4.13	13.04	$3.05x10^{-4}$	$1.97x10^{-2}$
JAML	1.01	4.39	12.97	$3.17x10^{-4}$	$2.02x10^{-2}$
CD83	1.13	4.41	12.2	$4.79x10^{-4}$	$2.68x10^{-2}$
RAC2	1.08	3.74	11.95	$5.45x10^{-4}$	$2.91x10^{-2}$
CCL2	1.16	3.89	11.42	$7.27x10^{-4}$	$3.53x10^{-2}$
TYMP	1.08	5.96	11.15	$8.41x10^{-4}$	$3.97\ x10^{-2}$
HAPLN3	1.1	3.7	10.46	$1.22x10^{-4}$	$4.88\ x10^{-2}$

* logFC = log fold change; logCPM = log counts per million; LR = likelihood ratio; P value = unadjusted p-value; FDR = FDR (false discovery rate)-adjusted p value

Supplementary Table 9 Downregulated differentially expressed genes between FFA cases and healthy controls (FDR ≤ 0.05 & |LFC| ≥ 1) *

Genes	logFC	logCPM	LR	P	FDR
HSD3B1	-3.12	4.66	29.45	5.74×10^{-8}	1.25×10^{-4}
HAO2	-3.00	3.79	22.28	2.36×10^{-6}	9.93×10^{-4}
FAR2	-1.86	6.84	20.87	4.9×10^{-6}	1.49×10^{-3}
MOGAT2	-2.42	3.60	20.88	4.91×10^{-6}	1.49×10^{-3}
DGAT2L6	-2.80	5.41	20.13	7.24×10^{-6}	1.89×10^{-3}
CYP4F8	-3.30	5.05	19.99	7.8×10^{-6}	1.96×10^{-3}
AWAT2	-3.11	5.82	19.58	9.67×10^{-6}	2.33×10^{-3}
PDZK1	-1.82	4.29	19.29	1.12×10^{-5}	2.52×10^{-3}
FADS2	-2.69	9.01	18.73	1.5×10^{-5}	2.97×10^{-3}
AADACL3	-3.11	7.44	18.18	2.01×10^{-5}	3.71×10^{-3}
CDKL2	-1.34	2.57	18.08	2.11×10^{-5}	3.72×10^{-3}
AWAT1	-2.51	4.44	16.97	3.8×10^{-5}	5.46×10^{-3}
PM20D1	-2.83	6.83	16.89	3.95×10^{-5}	5.46×10^{-3}
PNLDC1	-2.37	3.12	16.55	4.73×10^{-5}	5.92×10^{-3}
PDK4	-1.05	7.15	16.04	6.2×10^{-5}	7.15×10^{-3}
MSMO1	-1.08	7.06	15.50	8.23×10^{-5}	8.52×10^{-3}
SEC14L6	-2.51	6.18	15.14	9.99×10^{-5}	9.42×10^{-3}
TMEM56	-1.34	6.09	14.92	1.12×10^{-4}	1.01×10^{-2}
SEC14L4	-1.98	3.00	14.78	1.21×10^{-4}	1.06×10^{-2}
ACSBG1	-2.08	7.07	14.58	1.34×10^{-4}	1.13×10^{-2}
HSD11B1	-1.11	4.84	14.39	1.48×10^{-4}	1.21×10^{-2}
FADS1	-1.98	7.31	14.22	1.63×10^{-4}	1.32×10^{-2}
INSIG1	-1.12	7.34	13.84	1.99×10^{-4}	1.49×10^{-2}
TMEM97	-1.05	5.65	13.69	2.16×10^{-4}	1.58×10^{-2}
KRT79	-2.01	8.63	13.62	2.23×10^{-4}	1.59×10^{-2}
GLDC	-2.68	4.81	13.16	2.86×10^{-4}	1.90×10^{-2}
ELOVL3	-1.60	6.74	12.44	4.20×10^{-4}	2.47×10^{-2}
PDE6A	-1.92	3.51	12.23	4.71×10^{-4}	2.65×10^{-2}
GABRA4	-1.57	3.93	11.48	7.04×10^{-4}	3.50×10^{-2}
ACSM3	-1.16	4.11	11.36	7.50×10^{-4}	3.62×10^{-2}
THRIL	-1.00	4.35	11.31	7.70×10^{-4}	3.70×10^{-2}
GAL	-2.16	3.64	11.11	8.58×10^{-4}	4.01×10^{-2}
ROS1	-1.94	4.37	10.93	9.45×10^{-4}	4.18×10^{-2}
SOAT1	-1.19	7.52	10.75	1.04×10^{-3}	4.54×10^{-2}
PNPLA5	-2.25	4.35	10.71	1.07×10^{-3}	4.60×10^{-2}
ALOX15B	-2.22	7.25	10.62	1.12×10^{-3}	4.67×10^{-2}
TMEM91	-1.11	5.11	10.43	1.24×10^{-3}	4.95×10^{-2}

* logFC = log fold change; logCPM = log counts per million; LR = likelihood ratio; P value = unadjusted p-value; FDR = FDR (false discovery rate)-adjusted p value

Supplementary Table 10 Gene-set enrichment analysis (GSEA) for GO pathways: upregulated gene sets ranked by significance (p<0.05, q<0.05)

GO Term	Immune gene set description	p	q
REACTOME IMMUNE SYSTEM (M1045)	Genes involved in innate immune response	0.004	0.019
NABA MATRISOME (M5889)	Ensemble of genes encoding extracellular matrix and extracellular matrix-associated proteins	0.016	0.037
REACTOME CYTOKINE SIGNALLING IN IMMUNE SYSTEM (M1060)	Genes involved in cytokine signalling in immune system	0.022	0.037

Supplementary Table 11 Gene-set enrichment analysis (GSEA) for GO terms: upregulated gene sets ranked by significance (p<0.05, q<0.05)

GO Term	Immune gene set description	p	q
IMMUNE SYSTEM PROCESS (GO:0002376)	Any process involved in the development or functioning of the immune system, an organismal system for calibrated responses to potential internal or invasive threats	8.72×10^{-5}	0.011
IMMUNE RESPONSE (GO:0006955)	Any immune system process that functions in the calibrated response of an organism to a potential internal or invasive threat	3.73×10^{-4}	0.026

Supplementary Table 12 Gene-set enrichment analysis (GSEA) for GO terms: downregulated gene sets ranked by significance (p<0.05, q<0.05)

GO Term	p	q
LIPID METABOLIC PROCESS	0.0006	0.032
OXIDATION REDUCTION PROCESS	0.001	0.032
OXIDOREDUCTASE ACTIVITY	0.001	0.032
ENDOPLASMIC RETICULUM PART	0.001	0.032
NUCLEAR OUTER MEMBRANE ENDOPLASMIC RETICULUM MEMBRANE NETWORK	0.001	0.032
CELLULAR LIPID METABOLIC PROCESS	0.001	0.032
ENDOPLASMIC RETICULUM	0.002	0.046
MONOCARBOXYLIC ACID METABOLIC PROCESS	0.002	0.046

References

1. Fertig, R., Tosti, A. Frontal fibrosing alopecia treatment options. *Intractable Rare Dis. Res.* **5**, 314-315 (2016).

2. Tziotzios, C., Stefanato, C.M., Fenton, D.A., Simpson, M.A., McGrath, J.A. Frontal fibrosing alopecia: reflections and hypotheses on aetiology and pathogenesis. *Exp. Dermatol.* **25**, 847-885 (2016).

3. Seegobin, S.D., Tziotzios, C., Stefanato, C.M., Bhargava, K., Fenton, D.A., McGrath, J.A. Frontal fibrosing alopecia: there is no statistically significant association with leave-on facial skin care products and sunscreens. *Br. J. Dermatol.* **175**, 1407-1408 (2016).

4. Tolkachjov, S.N., Chaudhry, H.M., Camilleri M.J., Torgerson RR. Frontal fibrosing alopecia among men: a clinicopathologic study of 7 cases. *J. Am. Acad. Dermatol.* **77**, 683-690 (2017).

5. Lobato-Berezo, A., March-Rodríguez, A., Deza, G., Bertolín-Colilla, M., Pujol, R.M. Frontal fibrosing alopecia after antiandrogen hormonal therapy in a male patient. *J. Eur. Acad. Dermatol. Venereol.* **32**, e291-292 (2018).
